# Using Hierarchical Linear Models to Examine Approximate Number System Acuity: The Role of Trial-Level and Participant-Level Characteristics

**DOI:** 10.3389/fpsyg.2018.02081

**Published:** 2018-11-12

**Authors:** Emily J. Braham, Leanne Elliott, Melissa E. Libertus

**Affiliations:** ^1^Department of Psychology, University of Pittsburgh, Pittsburgh, PA, United States; ^2^Learning Research and Development Center, University of Pittsburgh, Pittsburgh, PA, United States

**Keywords:** approximate number system, numerosity, math ability, surface area, convex hull, hierarchical linear model

## Abstract

The ability to intuitively and quickly compare the number of items in collections without counting is thought to rely on the Approximate Number System (ANS). To assess individual differences in the precision of peoples’ ANS representations, researchers often use non-symbolic number comparison tasks in which participants quickly choose the numerically larger of two arrays of dots. However, some researchers debate whether this task actually measures the ability to discriminate approximate numbers or instead measures the ability to discriminate other continuous magnitude dimensions that are often confounded with number (e.g., the total surface area of the dots or the convex hull of the dot arrays). In this study, we used hierarchical linear models (HLMs) to predict 132 adults’ accuracy on each trial of a non-symbolic number comparison task from a comprehensive set of trial-level characteristics (including numerosity ratio, surface area, convex hull, and temporal and spatial variations in presentation format) and participant-level controls (including cognitive abilities such as visual-short term memory, working memory, and math ability) in order to gain a more nuanced understanding of how individuals complete this task. Our results indicate that certain trial-level characteristics of the dot arrays contribute to our ability to compare numerosities, yet numerosity ratio, the critical marker of the ANS, remains a highly significant predictor of accuracy above and beyond trial-level characteristics and across individuals with varying levels of math ability and domain-general cognitive abilities.

## Introduction

Without the use of symbols, counting, or formal mathematics, adults are able to rapidly estimate and compare the number of items in collections; we choose the bag of apples at the grocery store that contains the most apples, choose the parking lot that has the fewest cars, and stand in the check-out line that appears to have the fewest people. According to some researchers, the ability to intuitively compare approximate quantities taps into the Approximate Number System (ANS), a system in which we process numbers as noisy or imprecise magnitudes with overlap between neighboring representations of number (Dehaene, 1992; Barth et al., 2006). In the ANS, the degree of overlap between neighboring quantity representations increases for larger quantities and the discriminability between two numbers is determined by the numerical ratio between them. For example, quickly approximating if a bag with 11 apples has more than a bag with 10 apples is more difficult than quickly approximating if a bag with 11 apples has more than a bag with 7 apples. In addition, determining that 11 apples are more than 7 apples is as easy as determining that 22 apples are more than 14 apples. Thus, the critical marking of ANS processing is ratio-dependent performance (Dehaene, 1992).

To assess the acuity of children’s and adults’ ANS representations, researchers most frequently use non-symbolic number comparison tasks in which participants quickly choose the numerically larger of two arrays of dots over a series of trials that vary in the difficulty of the ratio between the two arrays. Across variations in temporal and spatial characteristics of the stimulus presentation, participants are generally faster and more accurate with relatively more disparate numerosities compared to less disparate ones (Dehaene, 1992; Cantlon and Brannon, 2006; Libertus et al., 2007; Halberda and Feigenson, 2008; Halberda et al., 2008; Soltész et al., 2010; Inglis et al., 2011; Dewind and Brannon, 2012; Price et al., 2012; Agrillo et al., 2013).

However, some researchers debate whether tasks designed to measure approximate number discrimination instead measure the ability to discriminate other perceptual variables that are confounded with number (Gebuis and Reynvoet, 2012; Leibovich et al., 2016; Henik et al., 2017). Here, we apply a novel analysis method, namely hierarchical linear modeling (HLM), to predict individual participants’ accuracy on each trial of a non-symbolic number comparison task from multiple trial-level characteristics (perceptual variables, presentation format) and participant-level controls (i.e., cognitive abilities such as visual-short term memory, working memory, and math ability) that are likely linked to performance on non-symbolic number comparison tasks. These analyses allow for greater specificity in unpacking the influence of several confounds simultaneously to account for differences in performance on the task both within and between individuals.

### The Role of Perceptual Variables for Non-symbolic Number Comparisons

In everyday life, number is frequently correlated with other visual characteristics (e.g., more apples take up more space). In non-symbolic number comparison tasks, non-numeric continuous dimensions of the dot arrays, such as cumulative area, cumulative perimeter, dot size, and/or visual density can influence judgments about numerosity (e.g., Allïk and Tuulmets, 1991; Durgin, 1995; Tokita and Ishiguchi, 2010; Dewind and Brannon, 2012). Researchers often attempt to rule out the use of these non-numeric continuous dimensions such that they are not consistently confounded with number throughout the entire experiment. However, these methods have been criticized for only manipulating a small subset of continuous magnitudes in any given trial, and thus allowing participants to use the other non-manipulated continuous magnitudes to predict numerosity (Gebuis and Reynvoet, 2012). For example, participants may use non-numerical visual cues such as convex hull or density to make numerosity judgments even when other visual features such as cumulative surface are not confounded with numerosity. Others have criticized this approach for not carefully accounting for all continuous dimensions (Clayton et al., 2015; Gilmore et al., 2016). For example, images from the freely available Panamath software^1^ are frequently used in the literature (Halberda and Feigenson, 2008; Halberda et al., 2008; Libertus et al., 2011, 2013a,b; Mazzocco et al., 2011; Libertus et al., 2012; Fazio et al., 2014; Hyde et al., 2014; van Marle et al., 2014; Haist et al., 2015; Norris et al., 2015; Patalano et al., 2015; Purpura and Logan, 2015; Bugden and Ansari, 2016; Norris and Castronovo, 2016; Braham and Libertus, 2017, 2018; Dillon et al., 2017; Lukowski et al., 2017; Geary et al., 2018), yet the software does not allow researchers to manipulate convex hull (i.e., the area of the smallest polygon that encompasses all of the dots in the set). Studies have demonstrated that convex hull is confounded with number in Panamath images, such that the more numerous set in each image typically also has a larger convex hull (Clayton et al., 2015; DeWind and Brannon, 2016). In a recent study, Gilmore et al. (2016) compared the influence of convex hull and cumulative surface area (which was highly correlated with dot diameter and density of the array) on both children’s and adults’ numerosity judgments on a non-symbolic comparison task. Convex hull information influenced accuracy across all age groups such that children and adults were more accurate on number comparisons when the convex hull ratio was large, but cumulative surface area information only influenced children’s, and not adults’, accuracy on number comparisons. These findings suggest that it is more difficult for adults to ignore convex hull information compared to cumulative surface area information.

Recent studies have used a new approach to constructing dot arrays that involves intentionally and systematically varying numerosity and non-numerical continuous dimensions in relation to one another in order to disentangle their influence on numerosity judgments (DeWind et al., 2015; DeWind and Brannon, 2016; Park et al., 2016; Starr et al., 2017). In these stimuli, features of the dot arrays are reduced to three parameters: number, size (i.e., the features related to individual element size, total surface area, and total perimeter), and spacing [i.e., the features related to convex hull and sparsity (convex hull/number of items)]. Using a modeling approach, DeWind et al. (2015) were able to dissociate the influence of the size and spacing features and show that while size and spacing bias adults’ numerosity judgments, the effect of these features was relatively small. Both children and adults primarily use number in numerical discrimination tasks, rather than size or spacing (DeWind et al., 2015; Starr et al., 2017). Further, there is evidence for earlier neural sensitivity to numerosity compared to these other continuous dimension features (Park et al., 2016).

### The Role of Spatial and Temporal Presentation Format for Non-symbolic Number Comparisons

Across studies that use non-symbolic number comparison tasks, there is also wide variation in the presentation format of the dot displays; some studies present the two arrays of dots simultaneously side-by-side, with spatial separation (i.e., one on either side of the screen or paired presentation), while other studies simultaneously present two arrays of different colors with spatial overlap (i.e., intermixed presentation). Most studies in the literature exclusively use either separated displays (e.g., Halberda and Feigenson, 2008; Piazza et al., 2010; Inglis et al., 2011; Lyons and Beilock, 2011; Libertus et al., 2012; Gilmore et al., 2013) or exclusively use overlapping displays (e.g., Dewind and Brannon, 2012; Halberda et al., 2012; Lourenco et al., 2012; Lindskog et al., 2013), with only a few studies using both presentation formats (Price et al., 2012; Norris and Castronovo, 2016). In a recent study using Panamath images, Norris and Castronovo (2016) directly compared different groups of participants’ accuracy on non-symbolic number comparison tasks using either spatially separated or spatially overlapping displays. Accuracy was higher and more reliable for participants who viewed the spatially separated displays compared to the overlapping displays. Lower performance on spatially overlapping displays may reflect the additional cognitive processing required to visually segment the arrays (Price et al., 2012).

A second major distinction in format across studies lies in the temporal aspects of the presentation. In the studies described above researchers presented the two spatially separated or spatially overlapping dot arrays simultaneously; however, a number of studies instead display the dot arrays sequentially, with one array followed by the other (Ansari et al., 2007; Hayashi et al., 2013). Smets et al. (2016) used a within-subjects design to directly compare participants’ performance on simultaneous trials, presented for 1500 ms, and sequential trials, in which each array was presented for 750 ms with a 500-ms pause between arrays. Participants had overall higher accuracy when arrays were presented simultaneously than when they were presented sequentially. There are a few potential explanations for these results. First, it has been suggested that additional working memory resources are required when the arrays of dots are presented successively (Price et al., 2012). Second, simultaneously presented side-by-side arrays may allow for more fine-grained, explicit comparisons of the two arrays than is possible on sequential trials in which only the second array can be kept in visual-spatial short-term memory (Brown and Rebbin, 1970; Smets et al., 2014, 2016). Thus, when images are presented sequentially, participants may use an alternative strategy in which they extract the numerosity of the first array to compare it to the numerosity of the second array (Frick, 1985; Smets et al., 2016).

These methodological differences in the spatial and temporal aspects of the dot displays are clearly present across studies yet infrequently accounted for in the literature. To our knowledge, only one study to date included all three presentation formats described above (simultaneously presented with spatial separation, simultaneously presented with spatial overlap, and sequentially presented) within a single study (Price et al., 2012). In a within-subjects design, Price et al. (2012) found significant positive correlations between participants’ performance in all formats of the task. In line with the findings of Norris and Castronovo (2016), participants’ performance was significantly worse on the simultaneously presented, spatially overlapping trials compared to the other two types of trials. However, unlike the results of Smets et al. (2016), there was no difference in participants’ performance on the simultaneously presented, spatially separated trials compared to the sequential trials. It is important to note that performance was measured using Weber fractions—an index of the imprecision of participants’ ANS representations—which has been shown to be a less reliable measure of ANS acuity compared to accuracy (Inglis and Gilmore, 2014). Nevertheless, together these findings suggest that performance on non-symbolic number comparison tasks is not independent of the spatial and temporal aspects of the presentation and that differences in accuracy across formats may be due to extraneous domain-general cognitive demands.

### The Link Between Non-symbolic Number Comparison Performance and Math Ability

Many studies propose a link between performance on non-symbolic number comparison tasks and measures of math ability, which involve using exact or symbolic representations of numbers to count and perform exact calculations (Halberda and Feigenson, 2008; Halberda et al., 2008, 2012; Gilmore et al., 2010; Inglis et al., 2011; Mazzocco et al., 2011; Libertus et al., 2011, 2012, 2013b; Dewind and Brannon, 2012; Lourenco et al., 2012; Bonny and Lourenco, 2013; Guillaume et al., 2013; Keller and Libertus, 2015; Braham and Libertus, 2017, 2018). These studies offer several potential explanations for the relation between the ANS and math. First, when children acquire knowledge of new symbolic numbers, they may map their new symbolic representations to their existing underlying ANS representations (Brankaer et al., 2014; Pinheiro-Chagas et al., 2014). Second, an intuitive understanding of approximate arithmetic with non-symbolic quantities may serve as a foundation for understanding symbolic arithmetic (Park and Brannon, 2014; Pinheiro-Chagas et al., 2014). And third, ANS representations may help facilitate error detection, as people with more precise ANS representations may more easily notice magnitude errors when performing symbolic calculations on a math assessment (Lourenco et al., 2012; Feigenson et al., 2013).

Although a number of meta-analyses provide support for the correlation between ANS acuity and math ability (Chen and Li, 2014; Fazio et al., 2014; Schneider et al., 2016), the correlations are overall low or moderate and there are many studies that report null or mixed results (Holloway and Ansari, 2009; Soltész et al., 2010; Castronovo and Göbel, 2012; Price et al., 2012; Fuhs and McNeil, 2013; Kolkman et al., 2013; Sasanguie et al., 2013). The discrepancy in findings across studies may be partly due to methodological differences in the way that math skills are assessed (Schneider et al., 2016; Braham and Libertus, 2018) or the way the non-symbolic number comparison task is constructed, including the spatial and temporal aspects of the presentation format and the controls for non-numerical continuous dimensions of the dot arrays (Norris and Castronovo, 2016). The inconsistent relation between ANS acuity and math ability across studies may also relate to participant-level characteristics of the sample, such as age (Inglis et al., 2011), individual differences in domain general cognitive skills that are needed across both tasks (e.g., working memory or inhibitory control; Fuhs and McNeil, 2013; Gilmore et al., 2013; Keller and Libertus, 2015), or other characteristics of the participants that often go unmeasured in these studies (e.g., math anxiety; Lindskog et al., 2017; Braham and Libertus, 2018).

### The Current Study

Although several studies have explored how specific trial-level characteristics, such as continuous magnitude dimensions or spatial and temporal presentation format, influence participants’ accuracy on non-symbolic number comparison tasks, less is known about how these variables operate uniquely from one another and potentially modulate numerosity ratio effects, a hallmark of non-symbolic numerical processing. In the present study, we used HLMs to predict people’s accuracy on the non-symbolic number comparison task from a comprehensive set of trial-level characteristics and participant-level controls. An advantage of this modeling approach is that it allows for the simultaneous estimation of the variation from person to person as well as from trial to trial. Here, we use a single model to simultaneously examine which features of the dot stimuli and which aspects of domain-general cognition relate to non-symbolic number comparison performance. We specifically address the following three research questions. First, how do trial-level characteristics, including numerosity ratio, spatial and temporal aspects of the presentation format, and continuous magnitude dimensions, and participant-level characteristics, including age, gender, math ability, phonological working memory, and visuospatial short-term memory, uniquely and independently relate to performance on individual trials of a non-symbolic number comparison task? Here we specifically focus on two continuous magnitude dimensions, cumulative surface area and convex hull, which are independent of each other and have been identified in the literature as potentially confounding variables (Gebuis and Reynvoet, 2012; DeWind and Brannon, 2016). As a robustness check, we also estimate these models with measures of average dot area and density included in the place of cumulative surface area and convex hull. Second, to what extent do these trial-level characteristics moderate the association between numerosity ratio and accuracy? Finally, to what extent does math ability moderate associations between these trial-level characteristics and individual’s accuracy on the non-symbolic number comparison task?

## Materials and Methods

### Participants

One-hundred thirty-five undergraduate students participated in a laboratory study in exchange for course credit. Three participants were excluded from all analyses due to incomplete data: two participants did not complete all measures of working memory and one participant did not report their gender. The final sample consisted of 132 participants (69 males) who ranged in age from 18 to 52 years of age (*M* = 19.71; *SD* = 4.23). The majority of our participants were in their first year of university (*n* = 83) and identified their race as White (*n* = 103). A subset of this sample completed a more extensive battery of tasks and those data have been previously reported elsewhere (Braham and Libertus, 2018).

### Measures

#### ANS Acuity

To measure ANS acuity, participants completed a total of 360 trials of a non-symbolic number comparison task in which they were presented with arrays of blue and yellow dots on a computer screen and instructed to select the color with more dots as quickly and accurately as possible. On all trials, participants indicated their response by pressing one of two keys on the keyboard, marked with either a yellow or a blue sticker. The correct response (i.e., the color with more dots) was counterbalanced across trials and participants received trial-level feedback—they heard a beep if they responded incorrectly.

**FIGURE 1:**
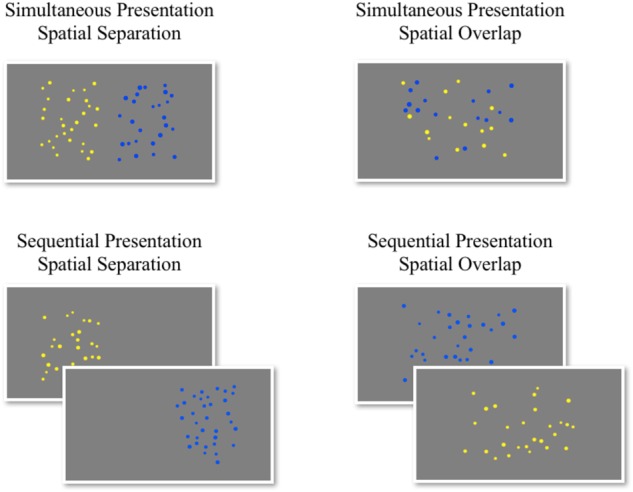
Example stimuli from each block of the ANS acuity task.

The 360 trials were divided into four blocks (90 trials per block) that varied in the spatial (spatial separation vs. spatial overlap) and temporal aspects (simultaneous vs. sequential presentation) of the stimulus presentation in an orthogonal design: (1) simultaneous presentation with spatial separation, (2) simultaneous presentation with spatial overlap, (3) sequential presentation with spatial separation, and (4) sequential presentation with spatial overlap (Figure 1). Participants completed the blocks in a counterbalanced order. All trials started with a fixation cross for 500 ms. On blocks with simultaneous presentation of the arrays, the blue and yellow dots appeared for 1500 ms; on blocks with sequential presentation of the arrays, one array appeared for 750 ms followed by the other for 750 ms. Participants could select their response on the keyboard either during the display of the dot arrays or during the blank screen that followed. Three participants were missing one (*n* = 2) or two (*n* = 1) blocks of this task but were retained in the analyses.

The images were presented using a custom-made Matlab script. All stimuli were extracted from the Psychological Assessment of Numerical Ability (Panamath)^2^. Each dot array contained between 12 and 36 dots and appeared on a gray background. Dot size varied within single arrays (average dot diameter = 36 pixels; allowed variation = 20%). The ratio of the larger quantity of dots to the smaller quantity of dots was evenly split across trials in one of five numerosity ratio categories (72 trials per ratio): 1.11, 1.14, 1.2, 1.25, 1.33. Surface area and convex hull ratios were calculated by dividing the value from the more numerous array by the value from the less numerous array. Surface area ratios ranged from 0.72 to 1.35. Convex hull ratios ranged from 0.72 to 1.71.

#### Math Ability

Participants’ math abilities were assessed using the Math Fluency subtest of the Woodcock Johnson III Tests of Achievement (Woodcock et al., 2001). Participants were presented with 160 simple addition, subtraction, and multiplication problems containing numbers in the 1–10 range (e.g., 8 – 0 = __; 3 × 6 = __). They were told to begin with the first problem, to work quickly and accurately, and to solve as many problems as they could within the 3-min time limit. The raw score (number of problems solved correctly) was converted into an age-normed standardized score with an expected mean of 100 and standard deviation of 15.

#### Visuospatial Short-Term Memory

We used a computerized flicker change detection task to assess participants’ visuospatial short-term memory capacity (Pailian and Halberda, 2015). On each trial, participants were presented with two arrays of yellow and blue dots on a gray background in continuous alteration. Each array flashed on the computer screen for 700 ms with a 900-ms pause between arrays. The two arrays were identical except for the color of one dot. Participants were told to search for the “target” dot (i.e., the dot that changed in color between the two images) as quickly and accurately as possible. They were instructed to press the space bar on the keyboard as soon as they detected the target to record their response time and to freeze the display, and then to use the computer mouse to click on the target dot to record their response. There were a total of 90 trials and the set size of the displays was manipulated across trials: 1/3 of the trials contained arrays with 6 dots, 1/3 of the trials contained arrays with 8 dots, and 1/3 of the trials contained arrays with 10 dots. Average response time on the correct trials, excluding trials in which participants’ response times were over two standard deviations from their average trial response time, was used as the measure of participants’ visual short-term memory with longer response times indicating smaller visual short-term memory capacity.

#### Phonological Working Memory

To assess phonological working memory, participants completed a backward digit span task, in which they listened to series of digit sequences presented at a rate of one item per second (e.g., “5, 9, 1, 3, 7”) and were instructed to recall the sequence in reverse order (e.g., “7, 3, 1, 9, 5”). The length of the sequences increased in difficulty throughout the task from three digits to 12 digits and participants were presented with two trials for each sequence length. Participant responses were marked as either correct or incorrect. Administration continued until the participant gave incorrect responses to both trials of the same sequence length. The length of the longest sequence in which the participant recalled at least one of the trials correctly was used as the participants’ phonological working memory span score.

### Procedure

All participants provided written, informed consent prior to participation. The study took place in a quiet laboratory room during a single 1-h session. Participants completed the tasks in the following order: ANS acuity, visuospatial short-term memory, phonological working memory, math fluency.

### Analysis Plan

A series of 2-level logistic hierarchical linear models (HLMs) were estimated to predict individual participants’ accuracy on each trial of the non-symbolic number comparison task (47,160 observations). These models predict accuracy on each trial of the task (1 = correct, 0 = incorrect). Trial-level characteristics, including numerosity ratio, surface area ratio, convex hull ratio, spatial presentation format (i.e., spatially separated vs. overlapping), and temporal presentation format (i.e., simultaneous vs. sequential) were included as level-1 predictors. Participant-level characteristics, including math fluency, age, gender, phonological working memory, and visuospatial short-term memory were entered at level-2 as predictors of level-1 intercept (i.e., individual’s average accuracy). Random intercepts by participant were included to account for individual differences in participants’ average accuracy across all trials. Descriptive statistics for all study variables, including trial-level characteristics as well as participant-level characteristics, are shown in Table 1.

**Table 1:** Descriptive statistics of level-1, trial-level characteristics (*N* = 360) and of level-2, participant-level characteristics (*N* = 132).

Level 1 Trial-Level Characteristics	*M* (*SD*)/%	Range
Numerosity Ratio (in raw values)	1.21 (0.08)	1.11, 1.33
Surface Area Ratio (in raw values)	1.01 (0.16)	0.73, 1.34
Convex Hull Ratio (in raw values)	1.12 (0.15)	0.83, 1.66
Dot Size Ratio (in raw values)	0.84 (0.14)	0.55, 1.02
Density Ratio (in raw values)	1.10 (0.15)	0.75, 1.61
Spatial Arrangement		
Overlapping	50%	
Separated	50%	
Presentation Format		
Sequential	50%	
Simultaneous	50%	

**Level 2 Participant-Level Characteristics**	***M* (*SD*)/%**	

Average Trial Accuracy	0.76 (0.05)	0.63, 0.89
Math Fluency	105.45 (13.17)	65, 153
Age (in years)	19.71 (4.23)	18, 52
Female	48%	
Visuospatial Short-Term Memory	1.10 (0.67)	0.27, 3.27
Phonological Working Memory	4.94 (1.40)	2, 8

First, main effects of trial-level characteristics on accuracy were estimated, controlling for participant-level characteristics. Surface area and convex hull ratios were natural log transformed, such that a surface area or convex hull ratio of 0 indicates that the continuous magnitude is equated across sets (as the untransformed ratio would be equal to 1), negative values indicate that the less numerous array had a larger value of this continuous magnitude, and positive values indicate that the more numerous array had a larger value of this continuous magnitude. Continuous indicators of surface area and convex hull were used in the analyses shown here as they offer more specificity regarding the degree to which continuous magnitudes are positively or negatively correlated with number^3^. Numerosity ratio was also centered at 1, such that a value of 0 indicates no difference in the two numbers (i.e., 1:1 ratio), and rescaled by a factor of 10, such that a one unit change in the rescaled variable represented a 0.1 unit change in ratio, for interpretability. Correlations among these transformed trial-level variables are shown in Table 2. All continuous level-2 variables were grand-mean centered.

**Table 2:** Correlations between trial-level characteristics for spatially separated and spatially overlapping trials.

	1	2	3	4	5
(1) Numerosity Ratio	–	– 0.01	– 0.37***	0.10	0.37***
(2) Surface Area Ratio	– 0.001	–	0.93***	0.14	– 0.13
(3) Dot Size Ratio	– 0.37***	0.93***	–	0.09	– 0.26*
(4) Convex Hull Ratio	– 0.02	– 0.07	0.07	–	– 0.89***
(5) Density Ratio	0.53***	– 0.06	– 0.25*	– 0.86***	–

*Correlations above the diagonal describe trials in which dot arrays were spatially separated (*n* = 90), whereas correlations below the diagonal describe trial in which arrays were spatially overlapping (*n* = 90). Correlations are based on the log-transformed surface area, dot size, convex hull, and density values. ^†^*p* < 0.10, *^∗^p* < 0.05, ^∗^*^∗^p* < 0.01, ^∗∗∗^*p* < 0.001.*

To answer our second research question regarding whether trial-level characteristics moderate associations between numerical ratio and accuracy, a series of interactions were then tested between numerical ratio and each additional trial-level characteristic. Interactions were first entered individually, and then all significant interactions were entered into a single model. Simple effects of numerical ratio predicting accuracy were then calculated at various levels of these moderating trial-level characteristics to probe significant interactions.

To answer our third research question regarding the role of math ability in these associations, we first included math ability as a level-2 predictor of level-1 intercept in order to address whether individuals with higher levels of math ability had higher overall accuracy on the non-symbolic number comparison task. Math ability was then included as a predictor of the ratio slope (i.e., as a cross-level interaction) to examine whether the magnitude of ratio effects differed across individuals with varying levels of math ability.

Finally, each of these models was estimated a second time with alternative measures of the perceptual variables described above. Specifically, raw cumulative surface area ratios were divided by the raw number ratio to represent the average dot size ratio of the larger set compared to the smaller set. Average dot size ratio ranged from 0.55 to 1.02. Additionally, raw number ratios were divided by the raw convex hull ratios to yield a ratio of the density of the larger set compared to the smaller set. Density ratio ranged from 0.75 to 1.61. Dot area ratio and density ratio were then natural log transformed and included as trial-level predictors in the place of surface area ratio and convex hull ratio respectively.

## Results

### Main Effects of Trial-Level and Participant-Level Characteristics

Results of models estimating main effects of trial-level and participant-level characteristics on individuals’ performance on the non-symbolic number comparison task are shown in the first column of Table 3. Numerosity ratio was a highly significant predictor of accuracy, as a 0.1 increase in numerical ratio (e.g., the difference between a 1.2 and 1.3 ratio) resulted in a 71% increase in the odds of correctly identifying the more numerous array. In other words, individuals were more accurate on trials in which the ratio of difference between the two arrays was larger, consistent with theoretical accounts of the ANS. Crucially, this association between numerosity ratio and accuracy was evident when controlling for continuous magnitude dimensions and variations in spatial and temporal presentation format of the task.

**Table 3:** Results of two-level logistic hierarchical linear models predicting trial-level accuracy on the non-symbolic number comparison task (1 = correct response) from trial-level and participant-level characteristics.

Fixed effects	OR (*SE*)	OR (*SE*)	OR (*SE*)
**Trial-Level Characteristics**			
Numerosity Ratio	1.71*** (0.03)	1.97*** (0.06)	1.98*** (0.06)
Surface Area Ratio	1.52*** (0.11)	3.30*** (0.80)	3.30*** (0.83)
Convex Hull Ratio	2.33*** (0.23)	2.98*** (0.87)	2.98*** (0.87)
Spatially Overlapping Presentation	0.66*** (0.02)	1.17* (0.08)	1.17** (0.08)
Sequential Presentation	1.12*** (0.02)	0.96 (0.06)	0.96 (0.06)
**Participant-Level Characteristics**			
Math Fluency	1.004* (0.002)	1.004* (0.002)	0.999 (0.003)
Age	0.99 (0.01)	0.99 (0.01)	0.99 (0.01)
Female	0.95 (0.05)	0.97 (0.05)	0.97 (0.05)
Visuospatial Short-Term Memory	1.003 (0.04)	1.003 (0.04)	1.003 (0.001)
Phonological Working Memory	1.02 (0.02)	1.02 (0.02)	1.02 (0.02)
**Trial-Level Interactions**			
Surface Area Ratio ^∗^ Numerosity Ratio		0.73** (0.07)	0.73** (0.07)
Convex Hull Ratio ^∗^ Numerosity Ratio		0.86 (0.12)	0.86 (0.12)
Spatially Overlapping Presentation ^∗^ Numerosity Ratio		0.74*** (0.02)	0.74*** (0.02)
Sequential Presentation ^∗^ Numerosity Ratio		1.09** (0.03)	1.09** (0.03)
**Cross-Level Interactions**			
Math Fluency ^∗^ Numerosity Ratio			1.003* (0.001)
Intercept	1.17*** (0.06)	0.90 (0.06)	0.90 (0.06)

**Random effect**	**Estimate** (***SE***)	**Estimate** (***SE***)	**Estimate** (***SE***)

Intercept	0.06 (0.01)	0.06 (0.01)	0.06 (0.01)

*Values shown in the table are odds ratios and their standard errors. Numerosity ratio was centered at 1, and surface area ratio and convex hull ratio were natural log transformed. Math fluency scores were mean-centered prior to estimating models. ^†^*p* < 0.10, *^∗^p* < 0.05, ^∗^*^∗^p* < 0.01, ^∗∗∗^*p* < 0.001.*

Furthermore, predicted accuracy significantly increased as surface area and convex hull ratios increased (i.e., as congruency between numerosity and surface area or convex hull increased). A one unit increase in convex hull congruency (i.e., the difference between trials in which convex hull was equal across sets, where this variable would be equal to 0, and trials in which the convex hull of the larger set was 2.72 times the size of the smaller set, where this variable would have a value of 1) resulted in a 133% increase in the odds of responding correctly, even when holding numerical ratio and other trial-level and participant-level characteristics constant. Similarly, a one unit increase in surface area ratio (i.e., the difference between trials in which cumulative surface was equal across sets, where this variable would be equal to 0, and trials in which the cumulative surface area of the larger set was 2.72 times the size of the smaller set, where this variable would have a value of 1) was associated with a 52% increase in the odds of responding correctly, controlling for numerical ratio and other trial-level and participant-level characteristics. Additionally, individuals tended to be more accurate on trials where arrays were presented with spatial separation (52% higher odds of responding correctly) and where arrays were presented sequentially (12% higher odds of correct response).

Few participant-level characteristics predicted level-1 intercepts at level-2. Math fluency scores were positively related to overall accuracy, such that a standard deviation increase in math fluency predicted a 7% increase in odds ratio. However, participant age, gender, phonological working memory, and visuospatial short-term memory were unrelated to overall accuracy in these models.

### Trial-Level Interactions With Numerosity Ratio

Interactions between trial-level characteristics and numerosity ratio were then entered into models individually. Surface area ratio, convex hull ratio, spatial presentation format, and temporal presentation format each significantly moderated associations between numerosity ratio and accuracy when included independently and as such were combined into a single model. Results are shown in the second column of Table 3. Significant associations remained for surface area ratio, spatial presentation format, and temporal presentation format.

**FIGURE 2:**
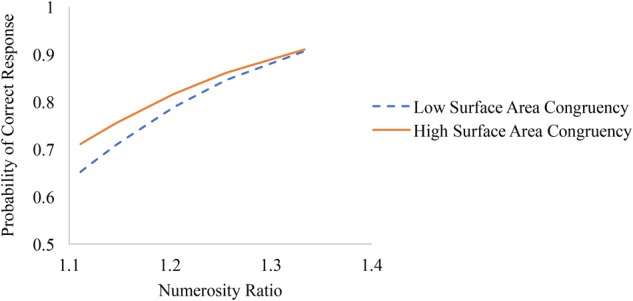
Associations between numerosity ratio and accuracy on trials with low congruency between surface area and numerosity (i.e., one standard deviation below 0, or a 1:1 ratio) and high congruency (i.e., one standard deviation above 0).

Numerosity ratio effects were significantly larger on trials where surface area ratio and numerosity ratio were less congruent (see Figure 2). In other words, the congruency between surface area and numerosity was most strongly related to accuracy on more difficult trials (i.e., trials with smaller numerosity ratio) and was not significantly related to performance on the easiest trials (i.e., trials with larger numerosity ratios).

**FIGURE 3:**
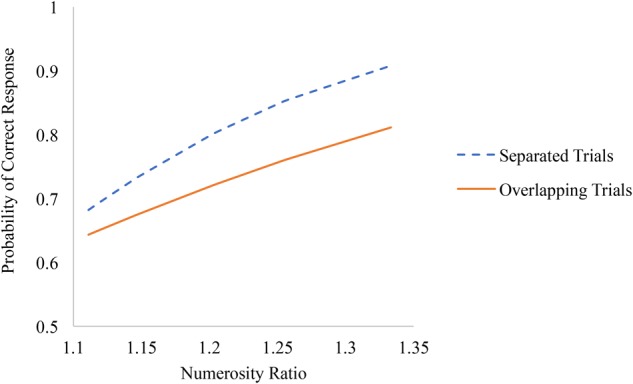
Associations between numerosity ratio and accuracy on spatially separated and overlapping trials of the non-symbolic number comparison task.

Additionally, numerosity ratio effects were significantly larger on spatially separated compared to overlapping trials (see Figure 3). The differences in accuracy between spatially separated and overlapping trials favoring separated trials were largest for easier trials (i.e., trials with larger numerosity ratios) compared to more difficult trials (i.e., trials with smaller numerosity ratios).

**FIGURE 4:**
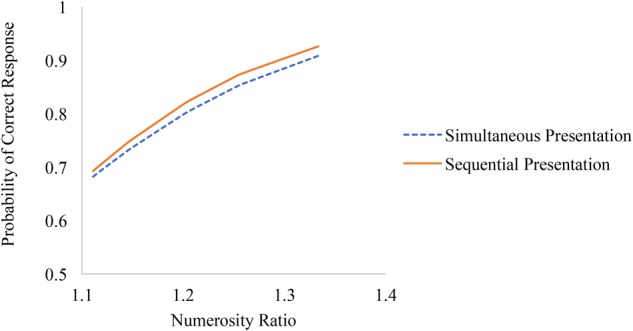
Associations between numerosity ratio and accuracy on simultaneously and sequentially presented trials of the non-symbolic number comparison task.

Finally, numerosity ratio effects were significantly larger on sequentially compared to simultaneously presented trials (see Figure 4). The difference in odds ratios among sequentially and simultaneously presented trials favoring sequential trials were largest among easier trials (i.e., trials with larger numerosity ratios) and were actually non-significant on the most difficult trials (i.e., trials with smaller numerosity ratios).

### Math Fluency Interactions

**FIGURE 5:**
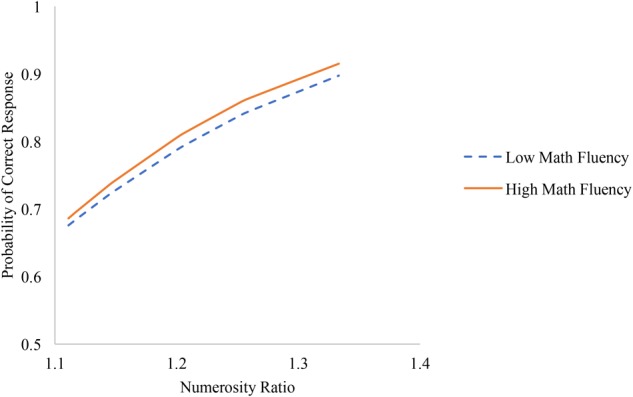
Associations between numerosity ratio and accuracy among individuals with low math fluency (i.e., one standard deviation below the mean) and high math fluency (i.e., one standard deviation above the mean).

Math fluency scores were then included as a predictor of the level-1 coefficient on numerosity ratio to represent a cross-level interaction between numerosity ratio and math ability. Model estimates are shown in the third column of Table 3. In addition to the positive main effects of math fluency on overall accuracy (i.e., intercepts), math fluency significantly predicted individuals’ numerosity ratio slopes, such that for participants with higher math fluency scores, associations between numerosity ratio and accuracy were higher (see Figure 5). Participants with higher math scores appear more responsive to number than participants with lower math scores. In other words, math fluency was more positively related to performance on easier trials (i.e., trials with larger numerosity ratios) but was not significantly related to performance on harder trials (i.e., trials with smaller numerosity ratios).

### Average Dot Size and Density as Trial-Level Predictors

Results from these models using measures of average dot size ratio and density ratio as predictors of accuracy are shown in Table 4. Consistent with the results described above, numerosity ratio remained a significant predictor of individuals’ performance across all model specifications. However, it is notable that both average dot size and display density were significant predictors of performance as well, as participants were more accurate on trials in which dot size congruency was higher and density congruency was lower. Dot size also significantly moderated numerosity ratio effects, such that numerical ratio effects were smaller on trials in which dot size was more congruent, consistent with the cumulative surface area interaction shown in Figure 2. Importantly, the inclusion of these alternative metrics of visual confounds in the stimuli did not change the remainder of the findings, including numerical ratio interactions with spatial or temporal presentation format or math fluency scores.

**Table 4:** Results of alternative two-level logistic hierarchical linear models predicting trial-level accuracy on the non-symbolic number comparison task (1 = correct response) from trial-level characteristics, including average dot size and density, and participant-level characteristics.

Fixed Effects	OR (*SE*)	OR (*SE*)	OR (*SE*)
**Trial-Level Characteristics**			
Numerosity Ratio	1.90*** (0.03)	2.06*** (0.07)	2.06*** (0.07)
Dot Size Ratio	1.52*** (0.11)	2.89*** (0.68)	2.89*** (0.68)
Density Ratio	0.43*** (0.04)	0.43** (0.11)	0.43** (0.11)
Spatially Overlapping Presentation	0.66*** (0.02)	1.21** (0.08)	1.21** (0.08)
Sequential Presentation	1.12*** (0.02)	0.96 (0.06)	0.95 (0.06)
**Participant-Level Characteristics**			
Math Fluency	1.004* (0.002)	1.004* (0.002)	0.999 (0.003)
Age	0.99 (0.01)	0.99 (0.01)	0.99 (0.01)
Female	0.97 (0.05)	0.97 (0.05)	0.97 (0.05)
Visuospatial Short-Term Memory	1.003 (0.04)	1.003 (0.04)	1.004 (0.04)
Phonological Working Memory	1.02 (0.02)	1.02 (0.02)	1.02 (0.02)
**Trial-Level Interactions**			
Dot Size Ratio ^∗^ Numerosity Ratio		0.77** (0.07)	0.77** (0.07)
Density Ratio ^∗^ Numerosity Ratio		1.03 (0.12)	1.03 (0.13)
Spatially Overlapping Presentation ^∗^ Numerosity Ratio		0.73*** (0.02)	0.73*** (0.02)
Sequential Presentation ^∗^ Numerosity Ratio		1.09** (0.03)	1.09** (0.03)
**Cross-Level Interactions**			
Math Fluency ^∗^ Numerosity Ratio			1.003* (0.001)
Intercept	1.20*** (0.06)	1.04 (0.08)	1.03 (0.08)

**Random effect**	**Estimate** (***SE***)	**Estimate** (***SE***)	**Estimate** (***SE***)

Intercept	0.06 (0.01)	0.06 (0.01)	0.06 (0.01)

*Values shown in the table are odds ratios and their standard errors. Numerosity ratio was centered at 1, and dot size ratio and density ratio were natural log transformed. Math fluency scores were mean-centered prior to estimating models. ^†^*p* < 0.10, *^∗^p* < 0.05, ^∗^*^∗^p* < 0.01, ^∗∗∗^*p* < 0.001.*

## Discussion

Issues surrounding (1) the measurement of the ANS and (2) the relation between individual differences in ANS acuity and math performance are both highly debated (Gebuis et al., 2016; Leibovich and Ansari, 2016; Leibovich et al., 2016). To our knowledge, we are the first to utilize hierarchical linear models (HLMs) to study the ANS and to simultaneously examine differences in non-symbolic number comparison performance from person to person and from trial to trial. This approach allowed us to account for the nested structure of our data, to account for variance in trial-level and participant-level variables at the same time, and to learn the distribution of effects across people by modeling the participant-level characteristics as random effects rather than fixed effects. Below we discuss our findings regarding the role of numerosity ratio, perceptual continuous dimensions, presentation format, and participants’ math ability on non-symbolic number comparison trial-level accuracy, and the role of these variables in modulating numerosity ratio effects.

### Effects of Numerosity Ratio

Replicating numerous studies (Dehaene, 1992; Cantlon and Brannon, 2006; Libertus et al., 2007; Halberda and Feigenson, 2008; Halberda et al., 2008; Soltész et al., 2010; Inglis et al., 2011; Dewind and Brannon, 2012; Price et al., 2012; Agrillo et al., 2013), we found that participants were more accurate on trials with easier numerosity ratios compared to more difficult numerosity ratios, i.e., they were more likely to correctly identify the larger quantity as the relative difference between the two numerosities became larger. Importantly, numerosity ratio was a highly significant predictor of accuracy above and beyond all measured trial-level variables, including convex hull ratio, surface area ratio, average dot size ratio, density ratio and variations in spatial and temporal presentation format of the stimuli. Thus, our finding is in line with prior work that suggests number, or numerosity ratio, is a highly salient dimension of non-symbolic stimuli (Cordes and Brannon, 2009; Libertus et al., 2014; DeWind et al., 2015; Park et al., 2016; Starr et al., 2017). Our study also extends this work by additionally controlling for participant-level variables, including participants’ age, gender, visuospatial short-term memory, phonological working memory, and math ability. Numerosity ratio remained a highly significant predictor of accuracy above and beyond all measured participant-level variables. These findings are particularly noteworthy given recent evidence indicating that critical non-numerical cues such as convex hull are not controlled for in the stimulus design of Panamath (Clayton et al., 2015). Importantly, numerosity ratio also remained a significant predictor of accuracy on all trial types (although not equally so, as will be discussed below), demonstrating that across task specifications, numerical information is related to performance. Thus, numerosity ratio, the critical marker of the ANS, seems to be an independent and robust indicator of non-symbolic number comparison performance.

### Effects of Continuous Dimensions on Non-symbolic Number Comparison

Our results indicate that our participants’ accuracy on the non-symbolic number comparison task cannot be explained entirely by numerosity ratio; certain trial-level characteristic of the dot arrays contribute to peoples’ ability to compare numerosities. On the one hand, the cumulative surface area of the dot arrays (or alternatively the average individual size of a dot in the arrays) was significantly associated with accuracy on the non-symbolic number comparison task, controlling for numerosity ratio and all other trial-level and participant-level characteristics. Specifically, increasing surface area congruency (the array with the larger number is also the array with the larger cumulative surface area), increased participants’ odds of responding correctly.

Cumulative surface area ratio and average individual dot size ratio also moderated the association between numerosity ratio and accuracy. On trials with easier numerosity ratios, participants performed similarly regardless of whether there was high surface area/dot size congruency or low surface area/dot size congruency, but on trials with more difficult numerosity ratios, participants were more accurate when there was high surface area/dot size congruency. While participants may be able to indicate the larger numerosity on easy trials by simply relying on numerosity as their primary cue, they may rely on other cues, namely surface area or dot size, to a greater extent as the numerosity ratio becomes more difficult to discriminate. When the numerosity ratio of the trial is difficult, using surface area or dot size provides a potentially useful, although not perfect, indicator that there are more items in the array, and leads to more accurate performance when the surface area or dot size information has high congruency with the numerosity information. This explanation is in line with the Signal Clarity Hypothesis, which states that the clarity of numerosity estimates can be supported by dimensions of continuous quantity when they co-vary with or are redundant with number (Cantrell and Smith, 2013; Cantrell et al., 2015). These findings are consistent with past work demonstrating that participants tend to be more accurate on surface area congruent trials compared to incongruent trials (e.g., Dewind and Brannon, 2012) but also extend this work by addressing how and when these congruency effects are likely to come into play.

On the other hand, increases in convex hull and density congruency also significantly predicted increases in accuracy. Participants were overall more accurate when the array with the larger number also had the larger convex hull or was denser, holding numerosity ratio and all other trial-level and participant-level characteristics constant. Increases in convex hull congruency were even more predictive of accuracy than increases in surface area congruency (133 and 52% increase in the odds of responding correctly, respectively). This result supports previous studies that describe the influence of convex hull on non-symbolic number comparison performance (Clayton et al., 2015; DeWind and Brannon, 2016) and those demonstrating that, for adults, convex hull may be a more salient dimension than surface area on these tasks (Gilmore et al., 2016). In contrast, density ratio was less predictive of accuracy than average dot size ratio possibly because extracting information about individual dot size may be easier than extracting information about cumulative surface area (Cordes and Brannon, 2008).

### Effects of Spatial and Temporal Variations in Stimulus Presentation Format

We also found a significant influence of both spatial separation and the temporal aspects of the stimulus presentation on participants’ accuracy. First, participants were more accurate on trials when the arrays were presented with spatial separation (52% higher odds of responding correctly) compared to spatial overlap, mirroring previous findings in the literature (Price et al., 2012; Norris and Castronovo, 2016). Together, these studies suggest that spatially overlapping displays are more difficult to compare, most likely because they require additional cognitive processing to visually segment the two arrays. Our study also provides new evidence that the spacing of the presentation format (separated or overlapping) moderates the association between numerosity ratio and participants’ accuracy, such that the benefit of spatially separated compared to spatially overlapping displays is greater on trials with easier numerosity ratios. One possible explanation for this result is that participants use different strategies when performing number comparisons of spatially separated and spatially overlapping arrays and that the use of these strategies is affected by numerosity ratio. However, future studies are needed to directly test this hypothesis.

Additionally, participants in our sample were significantly more accurate on trials when the arrays were presented sequentially compared to simultaneously. The benefit of sequential trials found here is opposite of the finding by Smets et al. (2016) who reported an advantage for simultaneously presented trials. It is possible that performance differences across the two studies are driven by presentation time differences; in both studies, each array in the sequentially presented trials was displayed for 750 ms, but Smets and colleagues had the arguably more difficult task because they included a 500-ms delay between the two arrays. It should also be noted that in our sample, the benefit of sequential trials over simultaneous trials was a relatively small effect (12% higher odds of responding correctly). Future studies manipulating this delay time would be instrumental in unpacking these findings and exploring how non-symbolic representations are maintained.

Mirroring the interaction we found for variations in spatial stimulus presentation with numerosity ratio, we found an interaction between temporal variations in stimulus presentation format and numerosity ratio. Participants showed greater benefit of sequential compared to stimultaneous presentation on trials with easier numerosity ratios. Again, one possible explanation for this result may be that participants use different strategies when performing number comparisons of sequentially and simultaneously presented arrays and that the use of these strategies is affected by numerosity ratio. One possible approach to test this hypothesis would be to use eye tracking to compare participants’ scanning patterns as they process the same arrays in the two conditions (see Pailian and Halberda, 2015, for a similar approach to compare differences between number and area comparisons). Another possible explanation is that the sequential presentation enables participants to form a solid representation of the first numerosity before comparing it to the second. However, this representational strength is more beneficial in an easy ratio when there is little overlap between the two representations of the numerosities.

### Effects of Participant-Level Characteristics

In addition to examining trial-level predictors of accuracy on this non-symbolic number comparison task, we were also interested in identifying participant-level predictors of individuals’ accuracy in this task. Consistent with past research (e.g., Halberda and Feigenson, 2008; Halberda et al., 2008, 2012; Inglis et al., 2011; Libertus et al., 2011, 2012; Mazzocco et al., 2011; Dewind and Brannon, 2012; Lourenco et al., 2012; Bonny and Lourenco, 2013; Guillaume et al., 2013; Keller and Libertus, 2015; Braham and Libertus, 2017, 2018), we found that participants with higher math fluency scores tended to have higher ANS acuity, as indicated by higher average odds of responding correctly. This association was quite small in magnitude (a standard deviation increase in fluency predicted a 7% increase in odds of correctly responding, which is equivalent to the difference between 60 and 62% probability) but was seen when controlling for domain-general cognitive skills.

Due to model specifications, math scores were included as a predictor of ANS performance rather than ANS acuity predicting math, as is typically seen in the literature (e.g., Gilmore et al., 2010; Libertus et al., 2011, 2013a; Mazzocco et al., 2011; Starr et al., 2013; Keller and Libertus, 2015). However, growing evidence indicates that these associations between math skills and the ANS may be bidirectional, such that math skills may actually support the development of the ANS. Piazza et al. (2013) demonstrated that adult speakers of Mundurukú, a language that lacks number words beyond five and therefore severely limits the mathematical concepts that speakers can articulate, have less precise representations of approximate quantities than do individuals from Western cultures who speak languages that include number words. Similarly, evidence with Western adults suggests that formal math education is associated with greater precision of the ANS (Nys et al., 2013; Lindskog et al., 2014). Furthermore, two recent studies utilized cross-lagged longitudinal designs have shown that children’s math skills predict later ANS acuity, even when controlling for earlier ANS acuity, suggesting that math may relate to changes in the ANS over time (authors, under review; Mussolin et al., 2014; but see He et al., 2016). As such, associations between the ANS and math may in fact be bidirectional, at least in early childhood. However, the present study was cross-sectional in nature, and so our findings cannot inform these hypotheses. Instead, our seemingly directional pathways simply reflect patterns of correlations across individuals.

Finally, we found that ratio effects on accuracy were moderated by math ability, such that individuals with higher math fluency were more responsive to ratio. These results indicate that individuals with stronger math skills may be more influenced by numerical information provided in the stimuli, although math ability did not significantly moderate associations between non-numerical information and accuracy, indicating that participants with stronger math skills did not necessarily rely on numerical information more and non-numerical information less. As such, more research is needed to unpack the ways that adults with varying levels of math skills process these displays and discriminate between quantities.

### Limitations and Conclusions

There are several limitations of this study that should be address in future research. First, unlike the methods of DeWind et al. (2015), we did not systematically vary surface area/dot size and convex hull/density ratios to have equivalent ranges. Thus, we acknowledge that our findings about the relative salience of numerosity ratio, cumulative surface area ratio, average dot size ratio, convex hull ratio, and density ratio, are constrained by the range of variability of these ratios in our stimuli. An important avenue for future research will be to combine the stimuli of Dewind and colleagues with our HLM analyses, which account for both trial-level and participant-level characteristics simultaneously. Second, our measure of participants’ math ability was limited to an assessment of speeded mental arithmetic. In light of work suggesting that ANS acuity may be differentially related to various aspects of math, and specifically that mental arithmetic may be more strongly related to ANS acuity than written arithmetic (Schneider et al., 2016; Braham and Libertus, 2018), future research is needed to follow up on this analysis approach using varied and more broad measures of math ability.

To summarize, our results indicate that certain trial-level confounds of the dot arrays, including cumulative surface area, average individual dot size, convex hull and density as well as spatial and temporal variations of the stimulus presentation, and certain characteristics of the participants, namely math ability, contribute to the ability to compare numerosities on the non-symbolic comparison task. Yet numerosity ratio, the critical marker of the ANS, remained a highly significant predictor of accuracy even when all other trial-level and participant-level characteristics were included in our models. Thus, our findings add further support for the argument that, although some trial-level confounds affect number judgments, numerosity ratio seems to be an independent and critical feature of non-symbolic number comparison performance, even across individuals with varying levels of math ability and domain-general cognitive skills.

## Ethics Statement

This study was carried out in accordance with the recommendations of the University of Pittsburgh’s Institutional Review Board. The protocol was approved by the University of Pittsburgh’s Institutional Review Board (PRO13090407). All subjects gave written informed consent in accordance with the Declaration of Helsinki.

## Author Contributions

EB and ML conceptualized the study. EB collected the data. LE and EB analyzed the data and drafted the manuscript. All authors contributed to editing, reviewing, and approving the final manuscript.

## Conflict of Interest Statement

The authors declare that the research was conducted in the absence of any commercial or financial relationships that could be construed as a potential conflict of interest.

## Footnotes

1 www.panamath.org

2 www.panamath.org

3 Trials could also be categorized as congruent (i.e., the array with the larger number had the larger cumulative area or convex hull), equated (i.e., the arrays had equal cumulative area or convex hull), and incongruent (i.e., the array with the smaller number had the larger cumulative area or convex hull). Models using these categorical indicators of congruency instead of the continuous ones yielded similar results to the ones described in the text.
